# Neoadjuvant immunochemotherapy for pulmonary large-cell neuroendocrine carcinoma: case report

**DOI:** 10.1186/s13019-024-02695-x

**Published:** 2024-04-15

**Authors:** Chang Xu, Guangyin Zhao, Hongyu Zhang, Di Ge, Jie Gu

**Affiliations:** Department of Thoracic Surgery, Zhongshan Hospital, Fudan University, Shanghai, 200032 China

**Keywords:** pLCNEC, Neoadjuvant immunochemotherapy, MPR, Case report

## Abstract

**Background:**

Pulmonary large-cell neuroendocrine carcinoma (pLCNEC) represents a rare malignancy characterized by its aggressive behavior and a notably high recurrence rate. Remarkably, there is currently no established standard treatment protocol for this condition.

**Case description:**

In this report, we present an intriguing case of pLCNEC diagnosed at clinical-stage IIB. This case involves a 64-year-old man with a smoking history spanning four decades. In our approach, we initiated a course of neoadjuvant chemotherapy in combination with pembrolizumab, administered for two cycles prior to surgical resection. This innovative treatment strategy resulted in a significant pathological response, culminating in a major pathological remission (MPR). As of the time of composing this report, the patient has been diligently monitored for 39 months post-surgery, exhibiting no indications of recurrence, and has demonstrated exceptional tolerance to the entire treatment regimen.

**Conclusions:**

We have first reported a clinically successful case of neoadjuvant combination chemotherapy with pembrolizumab in the treatment of pLCNEC. This case offers promising clinical insights and suggests that this therapeutic approach could be a viable option for managing pLCNEC.

## Background

A new classification within the spectrum of pulmonary neuroendocrine tumors, pulmonary large-cell neuroendocrine carcinoma (pLCNEC), was originally proposed by Travis et al. in 1991 [1]. The incidence of pLCNEC is 2.1–3.5% of all lung cancer cases. Currently, a universally accepted standard treatment regimen for pLCNEC is notably absent, with surgery being recommended as the primary approach for patients deemed suitable for resection. The 5-year survival rate for surgically managed patients ranges only from 13 to 57% [2]. Moreover, the landscape of neoadjuvant therapy for unresectable pLCNEC remains ill-defined, primarily due to the limited availability of data. Nevertheless, some studies have revealed that neoadjuvant chemotherapy shows great potential in the treatment of pLCNEC [2–4]. In this report, we share a compelling clinical case of pLCNEC, demonstrating a major pathological remission (MPR) following neoadjuvant combined chemotherapy with pembrolizumab.

## Case presentation

A 64-year-old man, who had been a smoker for four decades, was admitted to the hospital in January 2020 after the discovery of pulmonary space-occupying lesions during a routine physical examination three months earlier. An enhanced chest computed tomography (CT) scan revealed a large mass, measuring up to 36*28 mm in its maximum cross-section, along with a small, solitary nodule with a diameter of 9 mm in the right lower lobe. Subsequent Positron Emission Tomography (PET)-CT imaging disclosed that the large mass exhibited malignancy, with a standardized uptake value (SUV) of 20.2, while the smaller nodule was identified as a satellite lesion with an SUV of 2.3. No enlarged lymph nodes or distant metastases were detected. The patient underwent CT-guided percutaneous biopsy. Immunohistochemical analysis revealed negativity for thyroid transcription factor 1, napsin A, synaptophysin, CD56, protein 40, and protein 63, but positivity for CK7, a Ki-67 index of 80%, with only partial positivity for chromogranin A. Two experienced pathologists were invited to review the section of the medical record and found the morphology was consistent with the manifestation of large cell neuroendocrine carcinoma. Based on these findings, the patient was considerd as LCNEC at clinical stage IIB (cT3N0M0).

**Fig. 1:**
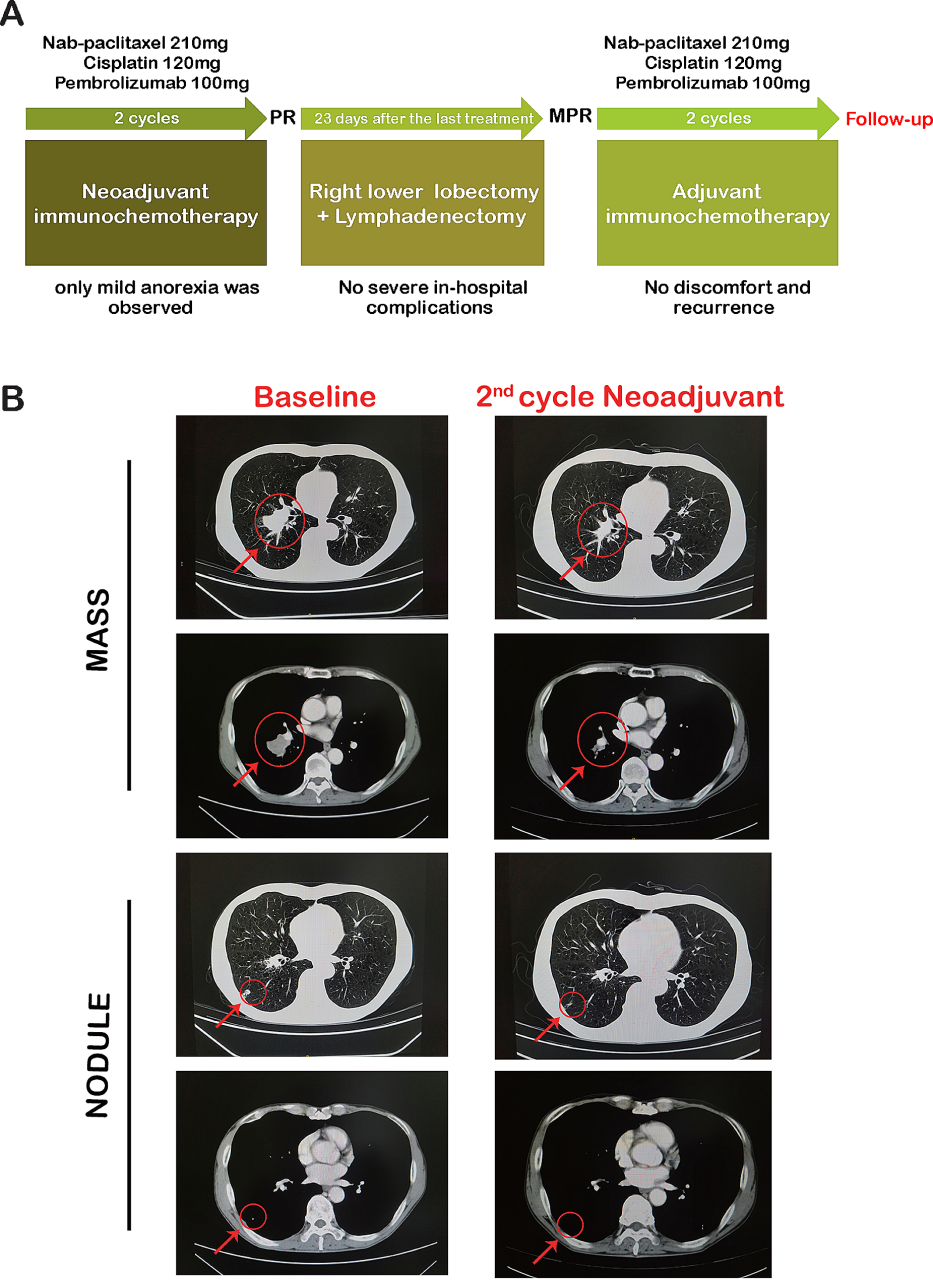
Disease evolution and treatment timeline under neoadjuvant immunochemotherapy. (**A**) The timeline therapy administration from the episode of treatment. PR, partial response; MPR, major pathological remission. (**B**) Computed tomography scan showed dynamic changes of lesions in radiology and revealed a partial response of disease after 2 cycles of immunochemotherapy

For treatment, we initiated neoadjuvant chemotherapy combined with pembrolizumab, administering two cycles at three-week intervals. The patient’s body surface area is about 1.6 square meters. The treatment regimen is paclitaxel 135 mg/m² + cisplatin 75 mg/m² + pembrolizumab 100 mg (Fig. 1A). Following these two cycles of immunochemotherapy, a reevaluation through enhanced chest CT indicated substantial reductions in both lesions, constituting a partial response (PR) according to efficacy assessment criteria (Fig. 1B). Subsequently, a right lower lobectomy and systemic lymphadenectomy were successfully performed 23 days after the last therapy (C2D1 + 23 days).

Postoperative pathology confirmed large-cell neuroendocrine carcinoma, with a major pathological remission (MPR) achieved, characterized by only a small amount of residual carcinoma tissue (< 5%) and a tumor bed size of 2.0 × 1.5 cm, accompanied by fibrous hyperplasia, inflammatory cell infiltration, and histiocytic reaction. Immunohistochemical staining corroborated the biopsy results, and no signs of lymph node metastases were identified. The TNM stage was downgraded to ypT1bN0M0 IA2. The patient was discharged just five days following the surgery without any significant in-hospital complications. The same immunochemotherapy regimen was administered for two additional cycles, and the patient exhibited only mild anorexia throughout the treatment, demonstrating excellent tolerance. To date, the patient has been followed up for 33 months and remains in excellent health, with no evidence of recurrence or metastases.

## Discussion and conclusions

Clinical data regarding pLCNEC are scarce due to its low incidence. The treatment landscape for LCNEC remains a subject of controversy. Notably, Rekhtman et al. proposed a molecular categorization of LCNEC into NSCLC-like LCNEC and SCLC-like LCNEC based on next-generation sequencing [5]. In the NSCLC-like LCNEC subgroup, mutations in KRAS, serine/threonine kinase 11 gene (STK11)/kelch-like ECH associated protein 1 gene (KEAP1), and TP53 were prevalent, while the SCLC-like LCNEC subgroup exhibited predominant RB1 and TP53 inactivation [6].

Sun et al. conducted research indicating that the SCLC-regimen displayed superior prognosis and objective response rates in advanced pLCNEC compared to the NSCLC-regimen. This observation aligns with the demonstrated efficacy of platinum compounds and taxanes in advanced disease [7]. Although surgery stands as the optimal treatment for early pLCNEC, some patients may derive benefits from neoadjuvant therapy, especially if surgical intervention is not feasible at the time of initial diagnosis. A single-center study reported a high response rate of 68% to platinum-based neoadjuvant therapy in 22 patients [3]. Another retrospective analysis pointed out that perioperative chemotherapy, including preoperative chemotherapy, may be beneficial in patients with resected LCNEC [4]. Saji H. also reported response rates of about 80% with neoadjuvant chemotherapy, indicating that neoadjuvant chemotherapy may favors survival [8]. Nevertheless, the role of neoadjuvant therapy in LCNEC treatment remains under active investigation.

Recent years have seen a phase II clinical study showing that neoadjuvant nivolumab could induce a major pathological remission (MPR) in 45% of resected tumors in NSCLC [9]. Furthermore, immunotherapy treatment, such as immunotherapy, has shown better overall survival and safety benefits versus docetaxel in NSCLC, based on results from OAK [10]. Despite the limited data available for pLCNEC, our case provides support for the positive influence of immune-neoadjuvant therapy and underscores the synergistic effect of neoadjuvant immunochemotherapy in pLCNEC. In our case, a clinical-stage IIB pLCNEC achieved a partial response (PR) after two cycles of neoadjuvant immunochemotherapy, and the patient underwent surgery with remarkable tolerance throughout the treatment. Postoperative pathology confirmed an MPR with less than 5% residual carcinoma tissue. The same immunochemotherapy regimen was continued for an additional two cycles after the operation. At the latest follow-up, the patient remained in good health with no signs of tumor recurrence. We hypothesize that chemotherapy-induced tumor cell death and the substantial release of tumor antigens triggered a robust and sustained response to immune checkpoint inhibitors (ICIs) through immune activation.

In conclusion, we report a clinically successful case of neoadjuvant combination chemotherapy with pembrolizumab in pLCNEC. We advocate for clinical trials to validate the clinical significance of neoadjuvant immunochemotherapy in pLCNEC. In summary, neoadjuvant immunochemotherapy may indeed represent a clinically viable option for the treatment of pLCNEC.

## Data Availability

The datasets used and/or analysed during the current study are available from the corresponding author on reasonable request.

## Abbreviations

IRB: Institutional Review Board
pLCNEC: pulmonary large-cell neuroendocrine carcinoma
MPR: major pathological remission
CT: computed tomography
PET: positron emission tomography
PR: partial response
